# Predicting protein linkages in bacteria: Which method is best depends on task

**DOI:** 10.1186/1471-2105-9-397

**Published:** 2008-09-24

**Authors:** Anis Karimpour-Fard, Sonia M Leach, Ryan T Gill, Lawrence E Hunter

**Affiliations:** 1 Center for Computational Pharmacology, University of Colorado School of Medicine, Aurora, Colorado 80045; 2 Department of Chemical and Biological Engineering, University of Colorado, Boulder, CO 80309; 3 Department of Electrical Engineering (ESAT), Research Division SCD, Katholieke Universiteit Leuven, B-3001 Leuven Belgium

## Abstract

**Background:**

Applications of computational methods for predicting protein functional linkages are increasing. In recent years, several bacteria-specific methods for predicting linkages have been developed. The four major genomic context methods are: Gene cluster, Gene neighbor, Rosetta Stone, and Phylogenetic profiles. These methods have been shown to be powerful tools and this paper provides guidelines for when each method is appropriate by exploring different features of each method and potential improvements offered by their combination. We also review many previous treatments of these prediction methods, use the latest available annotations, and offer a number of new observations.

**Results:**

Using *Escherichia coli *K12 and *Bacillus subtilis*, linkage predictions made by each of these methods were evaluated against three benchmarks: functional categories defined by COG and KEGG, known pathways listed in EcoCyc, and known operons listed in RegulonDB. Each evaluated method had strengths and weaknesses, with no one method dominating all aspects of predictive ability studied. For functional categories, as previous studies have shown, the Rosetta Stone method was individually best at detecting linkages and predicting functions among proteins with shared KEGG categories while the Phylogenetic profile method was best for linkage detection and function prediction among proteins with common COG functions. Differences in performance under COG versus KEGG may be attributable to the presence of paralogs. Better function prediction was observed when using a weighted combination of linkages based on reliability versus using a simple unweighted union of the linkage sets. For pathway reconstruction, 99 complete metabolic pathways in *E. coli *K12 (out of the 209 known, non-trivial pathways) and 193 pathways with 50% of their proteins were covered by linkages from at least one method. Gene neighbor was most effective individually on pathway reconstruction, with 48 complete pathways reconstructed. For operon prediction, Gene cluster predicted completely 59% of the known operons in *E. coli *K12 and 88% (333/418)in *B. subtilis*. Comparing two versions of the *E. coli *K12 operon database, many of the unannotated predictions in the earlier version were updated to true predictions in the later version. Using only linkages found by both Gene Cluster and Gene Neighbor improved the precision of operon predictions. Additionally, as previous studies have shown, combining features based on intergenic region and protein function improved the specificity of operon prediction.

**Conclusion:**

A common problem for computational methods is the generation of a large number of false positives that might be caused by an incomplete source of validation. By comparing two versions of a database, we demonstrated the dramatic differences on reported results. We used several benchmarks on which we have shown the comparative effectiveness of each prediction method, as well as provided guidelines as to which method is most appropriate for a given prediction task.

## Background

The number of published sequenced genomes has been growing in recent years, and at the present time, about 600 microbial genomes are fully sequenced. The next step after sequencing is to predict genes and their functions from the sequence. The explosion of sequence information has widened the gap between the number of predicted proteins and the number of experimentally characterized ones. *Escherichia coli *K12 is best characterized, but still has 15% of genes with unknown function [1]. Other genomes have between 15% and 70% uncharacterized genes.

The best established method for function prediction is based on sequence homology to proteins of known function. Unfortunately, strictly homology-based predictions are of limited use due to the large number of homologous protein families with no known function for any member [2-4]. Another way to assess the function of a sequence is through identification of its interactions with other proteins [5]. Interaction between proteins can be either physical or functional. In recent years, several computational methods for predicting protein-protein interactions have been developed. Machine learning methods [6-9] predict either functional relationships or physical linkages between proteins using a variety of information sources, including sequence information, expression data and localization data among others. Machine learning approaches typically require strong positive and negative set of examples, both of which may be difficult to obtain. Co-evolution methods [10-12] use phylogenetic trees to uncover potential protein linkages by identifying correlations in evolutionary histories. As such, these methods rely strongly on the availability and accuracy of phylogenetic trees. A third alternative to identify putative functional linkages between proteins are genomic context methods. The four major genomic context methods are: Gene cluster [13-15], Gene neighbor [16-18], and Rosetta Stone [19,20], and Phylogenetic profiles [21].

The Gene cluster (GC) method identifies operons within a microbial genome using intergenic distance as a predictor of operon structure [13-15]. Proteins belonging to the same operon are transcribed together, often because they have similar functional roles, participate in the same pathway, or bind to each other. Regardless of the nature of the linkage, we will refer to the relationship by the general term of 'functional linkage.' From a GC predicted operon, functional linkages can be inferred between adjacent genes. In contrast to GC for a single genome, the Gene neighbor (GN) method links genes that occur as chromosomal neighbors in multiple genomes thereby uncovering evolutionarily conserved operons [16-18]. The Rosetta Stone (RS) method predicts that distinct non-homologous genes have a functional linkage if their orthologs are fused in another organism [19,20].

The Phylogenetic profiles (PP) method predicts linkages among pairs of genes by determining whether they tend to be present or absent together in a set of reference genomes [21]. Previous studies evaluating the PP method have suggested ways to improve the prediction of protein-proteins linkages using variants of this method [22-27]. We have previously described how co-conservation networks (based on Phylogenetic profiles) vary with the selection of a reference group [25] and how co-conserved networks across well chosen sets of species can provide insight into the function of proteins that act in coherent biological processes [28]. We have also assessed the topological characteristics of bacterial co-conservation networks for the purpose of using such characteristics to improve protein function prediction [29].

Given the variety among these functional linkage prediction methods, the purpose of this paper is to provide insight into the tasks for which each method is most appropriate. Using first a benchmark of function prediction, we test whether each prediction method can uncover shared functional relationships among proteins. However, the various databases describing functional categories capture different aspects of biological relationships and the ability to predict those categories then depends on differences among the linkage prediction methods. The benchmarks are those commonly considered yet most studies simply report the overall coverage and accuracy of a prediction method for a given benchmark, rather than a breakdown by category. A notable exception is the study by Jothi *et al.*, (2007) which shows a difference in prediction accuracy over specific pathways when varying the set of reference genomes for a phylogenetic profile method [27]. Sun *et al*., (2007) showed that accuracy of predictions by phylogenetic profile method can be improved by using a set of genomes which are maximally distinct from one another [26]. The set of pre-compiled predicted protein linkages that were used in this analysis were obtained using all available organisms at their time of implementation [22]. We investigate which prediction method is most appropriate for a given category of proteins, a finding that is of interest to those who study a particular protein and might wonder from which resource to extract information about potential linkage. We recognize that the selection of the reference set can influence the phylogenetic profile results.

A second benchmark of reconstructing pathways evaluates whether the predicted linkages occur among proteins known to participate in the same biological process [20,21]. The pathway information was tested using EcoCyc [30] a carefully curated database of *E. coli *K12 pathways. Though the KEGG categorization also describes pathways, the EcoCyc resource is more specific and its sparser coverage does not permit the same analysis used for COG and KEGG. The ability to reconstruct an EcoCyc pathway was measured by counting the number of proteins in each known pathway which are connected by the predicted linkages to at least one other pathway member.

A third benchmark involves operon prediction using predicted linkages from the Gene cluster method. Previous studies have shown that operons tend to have short distances between their genes in bacteria [31,32]. Unfortunately, predictions based on intergenic distance alone aim to increase sensitivity so other sources of information must be added to bring the specificity to an acceptable level. Progress has been made toward a more generalized method for operon prediction based on a variety of diverse information sources, including codon usage statistics[33,34], and identification of promoter and terminator sequences [32,35,36]. However, very little has been done to examine the relative contribution of these features, individually and in combination, for operon prediction in genomes other than the genome(s) on which a prediction program is trained. In the few cases where cross-species application has been applied, the conclusions have been mixed [7,14]. The differences in portability of operon predictors might depend on the type and amount of information used for training, as suggested by Westover *et al*. 2005 [9]. We focus here on evaluating the most generally agreed upon feature, namely intergenic distance, alone and in combination with other features and investigate how the growing coverage in the databases themselves profoundly affects the reported accuracies.

We validate predicted Gene cluster functional linkages against known operons from *E. coli *K12 and *B. subtilis *because only these two organisms have a substantial number of experimentally verified operons. We examine whether features based on intergenic distance and protein function prove informative for both genomes. Moreover, we examine how combining linkages predicted by the other genomic context methods can improve Gene cluster operon prediction. We recognize that many of the methods being evaluated have been trained on *E. coli *K12 and *B. subtilis *data, so there may be some bias toward overestimation of accuracy when applied to other organisms. However, the general trends found are likely to hold up across a wide range of organisms.

Several studies have addressed the correlations between various genomic context and functional linkages [22,23,37-39]. Huynen *et al. *(2003) studied the correlation of individual linkages predicted by different genomic context methods in *Mycoplasma genitalium*. By using a combination of genomic context and homology search, they inferred new functional features for 10% of *M. genitalium *genes [37]. Other studies combined linkages from genomic context methods and their results showed that integrations of data outperform individual methods [38,39]. The STRING database provides a large collection of protein-protein linkages for a number of organisms from high-throughput experimental data, literature, and genomic context methods [39]. InPrePPI integrates protein linkages predicted by four genomic context methods and perform a systematic evaluation on these methods [38].

This study serves as a partial review of the many previous treatments of these prediction methods, yet offers a number of new observations. We use the latest available annotations such that our results represent an update to previous reports, allowing evaluation on a much larger set of annotations than available previously. Unlike previous studies which present overall conclusions on a number of benchmarks, we highlight the unique features of each genomic context method, with specific examples of where each method should best be applied, depending on the category of the protein(s) of interest. Also, beyond showing the concordance of functional characterization among linked proteins, we also provide an unbiased cross-validation study of function prediction methods which makes use of networks constructed from the linkages. By examining the performance of all four methods on the same benchmarks, we can provide potential explanations for the cause of the variable performance. Lastly, by presenting results on two versions of an annotation database, we can demonstrate how a changing benchmark might cause dramatic differences on reported results.

## Results

### Network topology using different sets of linkages

**Figure 1:**
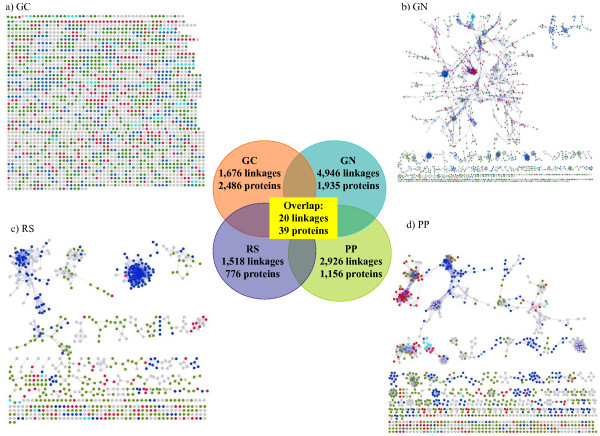
**Complete protein-protein networks of *E. coli K12***. Coverage and overlap are given in the central inset. Network proteins are color-coded based on the four KEGG functional categories: Unclassified (gray), Cellular processes (cyan), Environment information processing (blue), Genetic information processing (red), Metabolism (green) a) Gene cluster b) Gene neighbor c) Rosetta Stone d) Phylogenetic profile.

Protein functional linkages were extracted from the Prolinks database [22] and coverage of the number of linkages for the *E. coli *K12 genome varied depending on the confidence threshold (Table 1). Prolinks used all the available genomes (bacteria, archaea, and eukaryotes) at the time of their implementation. Previous studies have shown that accuracy of predictions can be improved by using a set of genomes which are maximally distinct from one another [25-27]. PP and GN offered the largest set of linkages at any threshold, with no linkages given at a high confidence level for GC. The large set of predicted linkages by GN is consistent with the previous report by Huynen *et al. *[37]. Among the set of 9,623 linkages predicted by any method at a threshold of 0.6 (used in all further results), there was very little overlap among the methods: 8,347 (87%) linkages appeared in only one method, 1,129 (12%) appeared in two methods, 127 (1%) appeared in three methods and only 20 (<1%) linkages were predicted by all methods (Table 1, Figure 1 center).

**Table 1:** Predicted linkages in E. coli K12 at different confidence levels in Prolinks v2.0

Method	Confidence > 0.4 Predicted #unique linkages/#unique proteins	Confidence > 0.6 Predicted #unique linkages/#unique proteins	Confidence > 0.8 Predicted #unique linkages/#unique proteins
GC	2,297/3,104	1,676/2,486	0/0
GN	11,789/2,843	4,946/1,935	625/396
RS	6,668/1,026	1,518/776	292/366
PP	10,029/1,730	2,926/1,156	1,308/779
			
Total	27,339/3,924	9,623/3,323	2,143/1,267
% predicted E. coli genes (4245 genes in NCBI)	92%	78%	30%

Number of overlapping linkages > 0.6			
	GC	GN	RS

GN	843	-	-
RS	100	202	-
PP	77	305	103

Protein-protein networks constructed from the predicted linkages also varied depending on the method used for prediction (Figure 1). The Gene cluster (GC) method, by definition, predicted small linear networks of proteins according to their gene order within an operon, while use of the remaining methods resulted in both small and large highly inter-connected clusters of proteins. Tight, functionally homogeneous clusters were seen in all graphs when colored by functional category.

The difference in network topology was quantified by a number of measures (Table 2). The clustering coefficient is defined as the edge density among the neighbors of a node [40]. The average clustering coefficient for the GN and PP networks was high. The RS network had a low clustering coefficient which suggested proteins were sparsely clustered. The methods showed an analogous trend in the average degree of connectivity, where PP and GN had similar high average connectivity. Sparse connectivity was found in RS (Table 2) indicating that there were fewer hubs (nodes with high connectivity) in this network.

**Table 2:** Topological analysis of different networks in E. coli K12.

Protein-protein linkage set	Average clustering coefficient	Average connectivity	Number of proteins
GN	0.56	5.11	1,935
RS	0.27	0.25	776
PP	0.83	5.06	1,156

### Functional Characterization of Interacting Proteins

Most proteins involved in the predicted linkages could be tested for concordance with either Kyoto Encyclopedia of Genes and Genome (KEGG) [41] categories or Clusters of Orthologous Groups (COG) functional categories [1]. The overall coverage was calculated as the percentage of proteins annotated by the source having at least one linkage predicted by a given method. Of the 2,587 proteins annotated to a COG category, the percent coverage per method was 69% (GC), 63% (GN), 39% (PP) and 26% (RS) while for the 1,936 proteins annotated to a KEGG category, the coverage was 67% (GC), 69% (GN), 40% (PP) and 27% (RS). The differences in percentages roughly followed the differences in the total number of proteins appearing in linkages for each method (Figure 1 inset), and the percentage of coverage of each method was similar whether using COG or KEGG categories.

**Figure 2:**
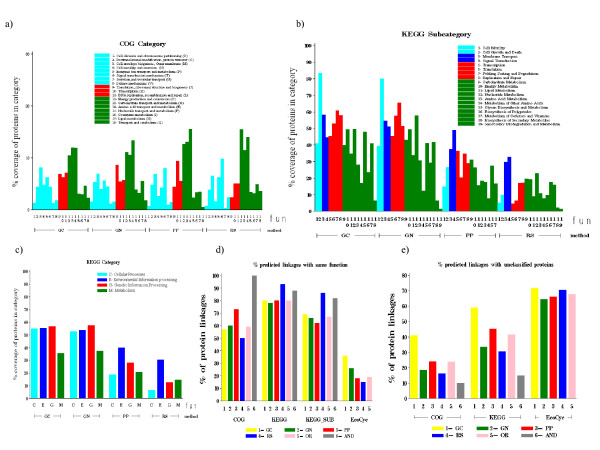
**Coverage and distribution of predictions relative to functional categorizations.** a) among all proteins annotated to a given COG category, the percent of those proteins with at least 1 linkage predicted by a given method b) percent using KEGG subcategories c) percent using KEGG subcategories d) percent of linkages predicted by a method where the linked proteins share the same function e) percent of linkages predicted by a method with at least 1 unclassified protein in the linked pair.

Only when examining these benchmarks by category did the two benchmarks show differences (Figure 2). Measuring concordance to each functional category as the percentage of proteins annotated to a category for which each method offered any linkages, the range of percent coverage of all methods was between 2–15% for COG, yet ranged from 2–85% for KEGG subcategory (Figure 2a–b). Moreover, GC and GN appeared to have much higher relative coverage than PP and RS for KEGG subcategories, while all methods had similar patterns of coverage for COG categories. This is consistent with the previous report by Sun *et al*. [38]. Given that the overall percent coverage of a given method was the same for COG as for KEGG as discussed above, we hypothesize that these differences were due to differences in the biological descriptions offered by these benchmarks. Thus, it is important to consider the methods at the level of the categories, not just the overall performance per benchmark, as has been done in most previous work.

When viewed per category, the methods were biased in their predictions toward proteins of a particular functional category. High coverage was observed in all methods for proteins annotated as signal transduction (KEGG 4, COG T), or membrane transport proteins (KEGG 3), whereas proteins involved in secretion and vesicular transport (COG U), biosynthesis of secondary metabolism (KEGG 18, COG Q) and xenobiotics biodegradation and metabolism (KEGG 19) had the least coverage in linkage predictions (Figure 2a–c). The abundance or lack of coverage has implications for later uses of the networks when studying proteins of these classes.

For particular classes of proteins, one or more prediction method showed better relative coverage for proteins. GN and GC were most effective at predicting linkages involving known proteins involved in folding sorting, degradation (KEGG 7, 65% and 60% of the proteins annotated to this category for GN and GC, respectively), and cell growth and cell death (KEGG 2, 80% and 83% respectively). These results suggest that many of the operons encoded in *E. coli *K12 may be highly biased toward proteins with these functional categories. Among the proteins considered by PP and RS, the most highly covered categories involved known signal transduction proteins (KEGG 4, 49% and 32% respectively) and known membrane transport proteins (KEGG 3, 37% and 30% respectively).

Concordance of predictions to annotation sources was then measured on the level of linkages rather than on the level of proteins (Figure 2d–e). The original paper describing the prediction methods reported that GN showed the most accurate and extensive coverage with respect to COG categories [22]. Our results serve as an update of those results. Despite the relatively higher coverage of GC and GN when measured at the level of proteins (Figure 2b–c), PP and RS generally showed stronger concordance by more often linking proteins assigned the same function than did GC or GN (Figure 2d). Nearly all 20 linkages found by all methods (AND) involved characterized proteins (Figure 2e) and most of the linkages linked proteins which had the same functional assignment (Figure 2d). For the uncharacterized proteins, GC had the highest percentage of linkages with at least one uncharacterized protein (Figure 2d) and hence could make predictions for some unknowns. Overall, the predicted linkages involving uncharacterized proteins from any of the methods are a valuable source of new hypotheses, as it is possible to infer function of unclassified proteins or gain a better understanding of the role of proteins through their connections with other proteins.

### Function Prediction Results

The function of an uncharacterized protein can be inferred from the functions assigned to its neighboring proteins in a linkage network. Overall, RS and PP networks proved most useful for function prediction. The quality of the linkage networks offered by each method was assessed using a cross-validation study of function prediction accuracy.

**Figure 3:**
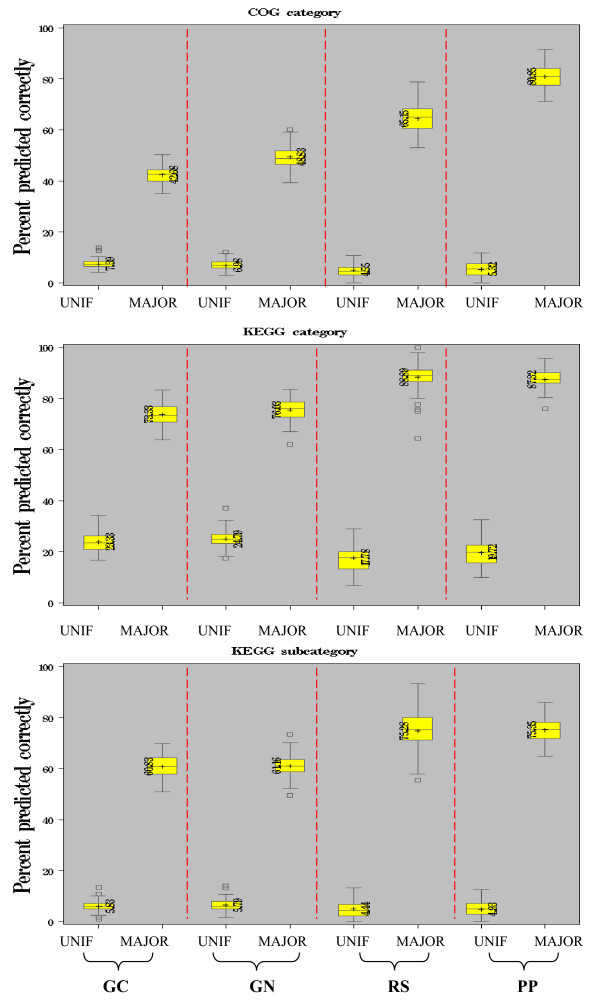
**Function Prediction Cross-evaluation of protein linkages for each method (PP, RS and GN):** UNIF: predicted function is sampled uniformly at random from the set of categories (KEGG 4 categories, COG 19 categories, KEGG 19 subcategories); MAJOR: predicted function is the majority assignment to immediate neighbors (ties are broken randomly).

The cross validation study was performed on proteins of known function by selectively hiding the function of one subset and predicting the function of the remaining proteins. Of the classified proteins with at least one classified neighbor in each network (GC: [2,033 and 1,206 proteins], GN: [1,729 and 1,213 proteins], PP: [946 and 715 proteins] and RS: [661 and 459 proteins] respectively according to COG and KEGG), 10% of the classified proteins were taken at random as the test set. Then these functions were hidden and predicted from quantities computed on the remaining 90% of classified proteins (training set). Functions were either assigned using a baseline method of assigning uniformly at random from among the protein categories (UNIF) or as the majority function of the immediate neighbors of a protein (MAJORITY). The percentage of correct predictions was recorded over 100 random partitions into training and test sets, using networks from the GC, GN, RS and PP methods (Figure 3). Proteins which had no classified neighbors as a result of creating the test set were not considered in the percent correct count.

Both the RS and PP methods showed the greatest increase over the baseline method, regardless of the annotation source. Interestingly, GN and GC showed lower performances despite often having comparable percentages of linkages with shared function (Figure 2e). This suggests that while the overall majority of linkages shared the same function in these networks, the majority of neighbors of a node did not, further highlighting the differences in topology of the individual networks.

#### Differences in Nature of Annotation Source

On the surface, the two functional annotation sources used here assessed similar notions of functional relationships, often sharing many similar categories (Figure 2 legends). Moreover, the general intuition behind each linkage prediction method involved an evolutionary tendency to optimize the cellular program by conserving sets of genes or increasing the proximity of related genes. It was therefore interesting that one method did not prove to be the best for both annotation sources. Overall, RS was individually best at preserving COG relationships while PP was best when using KEGG (Figure 2d). These results foreshadowed the relative differences in cross-validation performances (Figure 3), including the poor cross validation performance of GN which also had the lowest percentage of linkages sharing the same KEGG category.

Together with the observed differences in the category coverage results discussed earlier (Figure 2), these results suggested the two resources differed in how assignments were made to individual proteins. One possible explanation for the difference in COG and KEGG assignments may stem from the fact that COG functional assignments were made at the level of COG identifiers, whereby all members of the orthologous group were assigned the same set of COG functions [42]. It is well known that since COG identifiers were created from sequence homology, one disadvantage is that a COG identifier may associate paralogous genes. We investigated whether the existence of paralogs could explain the difference between RS and PP, in particular.

The potential existence of paralogs was investigated using the KEGG categorization among proteins assigned to COG ids. Of the 503 COG ids populated by *E. coli *K12 proteins among the RS and PP linkages, 361 had at least one protein annotated to a KEGG (sub)category. Of these, only 110 COG ids had the same KEGG function appearing among all annotated members, while only 93 COG ids had the same KEGG subcategory appearing among all members. That meant that nearly 66% of the annotated COG ids appearing among RS and PP linkages had at least one pair of proteins with distinct KEGG (sub)categories, suggesting that the original COG id assignments indeed grouped paralogous proteins.

The effect of paralogous proteins could therefore explain the difference in performance between RS and PP in preserving linkages among proteins in the same KEGG or COG functional category. Consider the argument that paralogous proteins are not likely to be fused (RS) yet might be present or absent concordantly (PP). This intuition was supported by the fact that among the predicted linkages from any method which were annotated by both functional sources, PP offered more of those linkages than RS where the COG function was the same for the linked proteins but the KEGG function was not (PP = 12%, RS = 5%, GC = 8%, GN = 8%). In fact, of all linkages offered by PP, 27% involve protein pairs assigned the same COG id (versus 10% of RS linkages, 8% of GC linkages and 0.4% of GN linkages). These results support the hypothesis that the improved performance of PP over RS on COG categories may be due to the ability of PP to detect paralogous genes.

#### Function Prediction from Combinations of Methods

Combining the sets of linkages from multiple prediction methods should allow for more accurate results than can be attained by any single method alone. Previous studies using different sets of linkages and other species have combined various sources such as mRNA co-expression, experimental data, literature, and phylogenetic profiles [39,43,44]. Until now two main approaches have been used to combine these data sets: 1) Reduce the number of possible relationships by looking at the overlap between data sources (AND case) or 2) Integrate all possible sources (OR case), often taking into account the reliability of each source. For the AND case, only 20 linkages were identified by all methods. In all of the predicted linkages, both proteins were assigned the same COG category if annotated. The OR case provided predictions for 9,632 linkages, but less than 59% of proteins involved in these linkages had annotations in COG.

Examples of integrating linkages from multiple methods (OR case) where the reliabilities of the methods vary include the STRING database [39] and the InPrePPI database [38], both of which probabilistically combine linkage confidences. The STRING database calculates a combined score for each pair of proteins using the assumption that the features from various sources are independent [39]. The InPrePPI database calculates the reliability of each of the four genomic context methods then optimally weights the score of each method and finally integrates the score. We considered two options: using reliabilities assigned to all linkages from a given method (Noisy-OR_meth_) or using reliabilities assigned per linkage based on the confidence level provided by the Prolinks database (Noisy-OR_conf_). Given a network created by integrating multiple sources of linkages, function predictions could be made by taking the majority function vote of the immediate neighbors as in the previous cross-validation study. However, in this case, varying reliability across linkages could be incorporated in the function prediction algorithm by instead taking a weighted vote among immediate neighbors, where each linkage was weighted proportionally to the reliability of that linkage.

**Figure 4:**
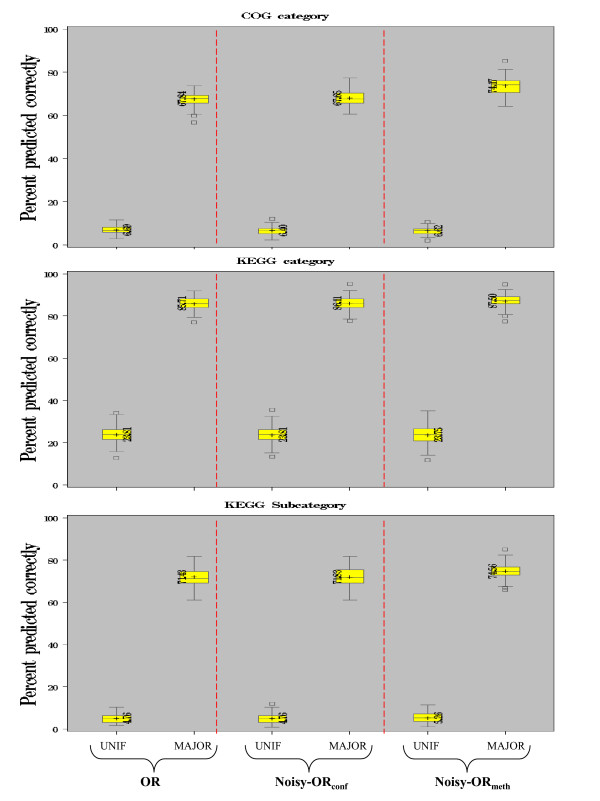
**Function Prediction Cross-evaluation of protein-protein linkages for method combinations (OR, Noisy-OR_conf _or Noisy-OR_meth_,):** UNIF: predicted function is sampled uniformly at random from the set of categories (KEGG 4 categories, COG 19 categories, KEGG 19 subcategories); MAJOR: predicted function is the (weighted) majority assignment to immediate neighbors (ties are broken randomly).

We were interested in identifying the most effective method among the three integration strategies, namely the unweighted OR graph and the two weighting strategies involving Noisy-OR (Figure 4). Under the same cross-validation scheme as before, the distributions of percentage correct predictions over the 100 random runs for each pair of strategies were compared using a standard t-test for equal means. Both of the weighted combination strategies, Noisy-OR_meth _and Noisy-OR_conf_, showed improvement over the unweighted OR with a slight preference for Noisy-OR_meth _(Noisy-OR_meth _vs OR p < 0.001 (COG, KEGG subcategory and p = 0.0049 (KEGG)) and Noisy-OR_meth _vs Noisy-OR_conf _p < 0.001 (COG, KEGG subcategory) and p = 0.0213 (KEGG)). These results suggested that assigning reliabilities per method was a more effective strategy than estimating edge-wise reliabilities.

While the performance of the combined network was similar to using RS or PP individually, the results were remarkable given that the same level of performance was seen with a great increase in coverage offered by the combination of networks (from predicting functions for nearly 776 proteins in the individual graphs to predicting 2,574 in the combined graph).

### Pathway reconstruction Results

**Figure 5:**
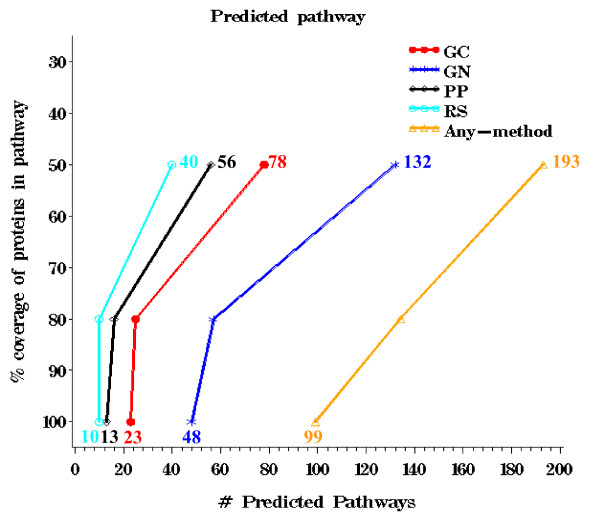
**Percentage of proteins in a pathway with at least one linkage in each method, using confidence threshold 0.6. **Numbers above and below each line represent 50% and 100% coverage, respectively.

The EcoCyc database [30] offered well curated descriptions of pathways, involving between 1 and 46 proteins per pathway. The granularity of the EcoCyc assignments resulted such low coverage (Figure 2d–e) that we could not treat this resource in the same manner as the COG and KEGG resources. Thus, we instead evaluated the EcoCyc benchmark in terms of pathway reconstruction. A pathway was considered to be completely reconstructed by a method if 100% of the proteins annotated to the pathway could be connected to at least one other member of the pathway through linkages predicted by the method. Of the 209 known (non-trivial) pathways documented in EcoCyc, we were able to reconstruct 99 complete pathways by at least one of the methods. The trehalose degradation II pathway (3 members) was the only pathway where no method contained linkages connecting at least two pathway members. The GN method was most effective at pathway reconstruction, detecting linkages among all proteins listed in 48 complete pathways (Figure 5). At least 50% of the proteins in 193 pathways were predicted to connect to the correct pathway by at least one method. The poor performance of PP relative to GC was especially surprising since PP predicted nearly twice as many linkages as GC and our definition of reconstruction favored more highly connected graphs.

### Operon Prediction Results

**Figure 6:**
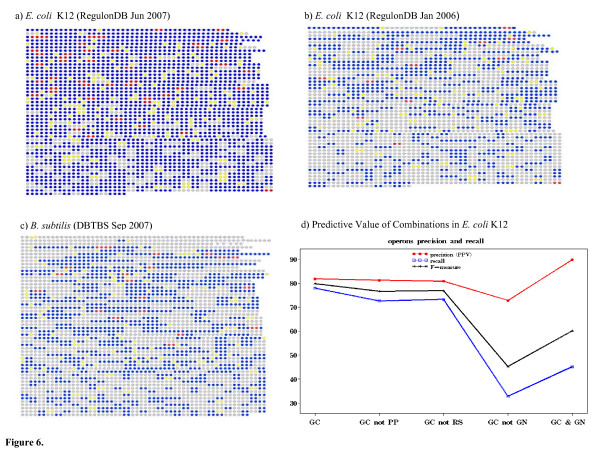
**Operon Prediction in *E. coli *K12 and *B. subtilis*.** a-c) Node coloring depicts operon status according to RegulonDB or DBTBS: both proteins in the same operon (blue nodes), both operon proteins but not in the same operon (red nodes), only one classified as an operon protein (yellow nodes), or both not classified as operon proteins (grey nodes) d) precision and recall using different combinations.

The accuracy of protein linkages predicted by Gene cluster (GC) were evaluated against known operons for *E. coli *K12 [45] and *B. subtilis *[46] (Table 3, Figure 6). For *E. coli *K12, the GC method predicted completely 489 out of 830 known operons (59%) listed in the June 2007 version of RegulonDB (Figure 6a). There were 152 operons in *E. coli *K12 that were missed completely by GC. We analyzed all of the missed operons and noted that some of the missed operons were the result of change in annotation based on the length of the intergenic region. The cases involving unclassified or non-operon proteins were of particular interest since they represented novel predictions. In fact, an increase in the number of linkages within operons (gray nodes become blue nodes) in the side-by-side comparison with an earlier release of RegulonDB (Table 3, Figure 6b) dramatically highlighted that many of the linkages predicted by GC had indeed later been recognized as correct operons by RegulonDB.

**Table 3:** Statistics on operon predictions.

	**E. coli K12**		**B. subtilis**
**Operon Database**	**RegulonDB** **Jun 2007**	**RegulonDB** **Jan 2006**	**DBTBS** **Sep 2007**
**Total # of known operons**	830	355	418
Completely predicted	489 (59%)	213 (60%)	333 (80%)
Completely missed	152 (18%)	52 (15%)	24 (6%)
			
**Total # of GC predicted linkages**	1676	1676	2104
Both proteins in same operon (Fig 6 blue nodes)	1282 (76%)	661 (39%)	843 (40%)
Both operon proteins, not same operon (Fig 6 red nodes)	46 (3%)	12 (1%)	20 (1%)
Only 1 classified as an operon protein (Fig 6 yellow nodes)	161 (10%)	101 (6%)	122 (6%)
Both not classified as operon proteins (Fig 6 grey nodes)	187 (11%)	902 (54%)	1119 (53%)
			
**Total # of GC predictions where both are operon proteins annotated with function**	1520 (EcoCyc Jun 2007)	1392 (EcoCyc Jan 2006)	1583 (DBTBS Sep07)
**Number of True Positives (same operon)**	1244	659	706
Mean (Median) intergenic distance in bases	20.68 (10)	22.04 (10)	28.98 (17)
Percentage with < 25 bases	74	73	63
Percentage with > 25 and < 50 bases	12	10	16
Percentage with > 50 bases	14	17	21
**Number of False Positives (diff operon)**	276	733	877
Mean (Median) intergenic distance in bases	69.29 (74)	38.79 (24)	43.80 (29)
Percentage with < 25 bases	15	51	46
Percentage with > 25 and < 50 bases	11	14	15
Percentage with > 50 bases	74	35	39

Analogously, linkages predicted by GC in *B. subtilis *were compared against known operons listed in the September 2007 version of the DBTBS database (Table 3, Figure 6c). The GC method predicted completely 333 out of 418 known operons. There were 24 missed operons. The decreased percentages (Table 3) reflected the sparser coverage of the DBTBS database relative to RegulonDB; in fact the percentages suggest the *B. subtilis *database is lagging one year behind that of *E. coli *K12.

#### Improving predictions using combinations of methods

The predictive performance of GC in *E. coli *K12 could be improved by comparing the linkages found with GC against those found by other methods. We considered a true positive (TP) when both proteins involved in a linkage had a functional annotation in EcoCyc and were known to reside in the same operon by RegulonDB and a false positive (FP) when at least one of the classified proteins was not listed by RegulonDB to be in the same operon. The Positive Predictive Value (PPV) of the set of GC linkages was calculated as PPV = TP/(TP+FP). Calculating the PPV depends on the completeness of the available sources. In fact, the PPV of GC linkages comparing two versions of RegulonDB showed an increase from 0.47 (Jan 2006) to 0.82 (Jun 2007). Thus, assessments of false positive linkages must be taken as provisional. Restricting the set of TP and FP linkages to be among only functionally annotated proteins was also meant to improve the chances that the false positives were indeed false.

Removing linkages in GC also predicted by PP or also predicted by RS resulted in no significant change in PPV (from 82% to 81%, Figure 6d). This suggested that distance between proteins was a more informative and decisive factor than co-conservation or gene fusion for prediction of operons. However, removing linkages from GC also predicted by the GN method showed a significant decrease in PPV (from 82% to 73%). This indicated that the additional support of observing neighboring genes in multiple genomes, as seen in the linkages common to GN, accounted for a large part of the information captured by GC linkages. Moreover, this suggested that linkages common to GC and GN were likely to be correct predictions. Considering only linkages predicted by both GC and GN resulted in an increase of PPV from 82% to 87%. Given the use of multiple genomes in PP, RS and GN, one might assume that operons missed when combinations of methods were used were specific to *E. coli *K12.

#### Improving predictions through analysis of intergenic distance

**Figure 7:**
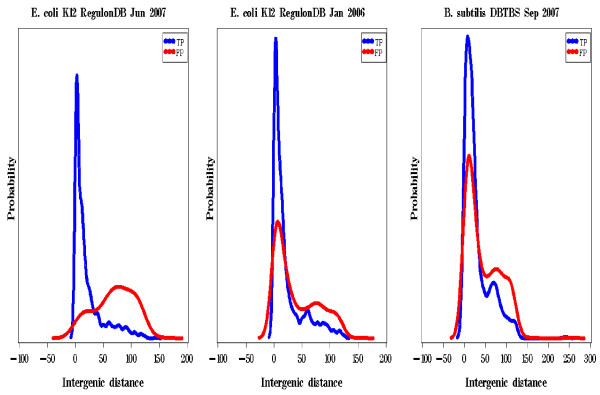
**Intergenic distance between gene pairs.** True positives (TP) count when when both proteins involved in a linkage had a functional annotation in EcoCyc (DBTBS) and were known to reside in the same operon by RegulonDB (DBTBS) and a false positive (FP) when at least one of the classified proteins was not listed by RegulonDB (DBTBS) to be in the same operon. a) *E. coli *K12 using RegulonDB June 2007 b) *E. coli *K12 using RegulonDB January 2006 c) *B. subtilis *using DBTBS September 2007.

Many different methods have proven to be valid predictors of operon structure (Table 4) yet most striking is that among all these studies, one of the most valuable predictors has simply been intergenic distance. We found that predicted operon linkage pairs with intergenic distances greater than 50 bases were more likely to be false positives (Table 4, Figure 7). In *E. coli K12*, 74% of the false positive linkage predictions involved proteins with intergenic distances >50 bases in contrast to only 14% of true positives with intergenic distances >50. The median intergenic difference also differed widely between the true positives (10 bases) and false positives (74 bases). A side by side comparison to an earlier version of RegulonDB (Figure 7a–b) demonstrated that many of the false positives in the earlier version with shorter intergenic distances were resolved to be true positives in the later version of RegulonDB.

**Table 4:** Table of previous operon prediction methods.

Predictor method	Data Types	Applied species	Operon Data	Sen (%)	Spe (%)	Accuracy
Probability Current study, from Bowers et al., 2004	Intergenic distance	E. coli K12	2684 TU E. coli K12 Jun07	78	_	_
		B. subtilis	(831 multiprotein operons)			(precision = PPV = 82% E. coli K12)
			1823 pairs in same operon			
			770 TU E. coli K12 Jan06	75	_	_
			(356 mulitprotein operons)			(precision = 47%)
			905 pairs in same operon	94	_	_
			1115 TU B. subtilis			(precision = 45% B. subtilis)
			(419 multiprotein operons)			
			972 pairs in same operon			
HMM Yada et al., 1999	Sequence information	E. coli K12	390 TU	59	_	_
Naïve Bayes Craven et al., 2000	Sequence information	E. coli K12	365 TU	75	91	83%*
Log likelihood Salgado et al., 2000	Intergenic distance, functional classes	E. coli K12	361 TU (237 multi)	88	88	82%* Distance
			572 pairs in same operon			88%* Both
			346 pairs at TU border			
Probability Ermolaeva et al., 2001	Conserved gene clusters across 34 genomes	E. coli K12	389 TU	48	92	70%*
			541 pairs in same operon			
			263 pairs at TU border			
			(pair if ≤ 200 bp apart)			
HMM Tjaden et al., 2002	Expression data	E. coli K12	463 pairs in same operon	63	99	81%*
Graph analysis Zheng et al., 2002	Metabolic pathway information	E. coli K12 (also applied to 42 other genomes)	128 TU metabolism related	89	87	88%*
Log likelihood Moreno-Hagelsieb et al., 2002	Intergenic distance	B. subtilis (trained on E. coli K12, applied to 68 genomes)	100 TU B. subtilis	88	88	88%* B. subtilis
			310 pairs in same operon			82%* E. coli K12
			123 pairs at TU border			
Bayesian posterior probability Sabatti et al., 2002	Intergenic distance, co-expression	E. coli K12	257 TU	82	70	76%* Co-expr
			604 pairs in same operon	84	82	83%* Distance
			151 pairs at TU border	88	88	88%* Both
Bayesian network Bockhorst et al., 2003	Intergenic distance, sequence information, expression data	E. coli K12	365 TU	78	90	84%*
Machine learning Romero et al., 2004	Intergenic distance, functional information	B. subtilis (trained on E. coli K12)	100 TU B. subtilis 446 TU E. coli K12	81	48	65%* B. subtilis
				87	86	87%* E. coli K12
				(91)	(87)	(89%* if use all info on E. coli)
Bayesian classifier De Hoon et al., 2004	Intergenic distance, operon length, gene expression	B. subtilis	635 TU	82^¶^	89^¶^	83^¶ ^distance
			582 pairs in same operon	80^¶^	79^¶^	80^¶ ^expression
			91 pairs at TU border	88^¶^	88^¶^	89^¶ ^all
Machine learning without extensive training data Westover et al.,2005	Intergenic distance, functional classes, conserved gene clusters	E. coli K12 (validated by known operons)	E. coli K12:	88	80	84%* E. coli K12
		B. theta (validated by co-expression)	797 pairs in same operon			
			294 pairs at TU border			
			B. theta:	73	80	76.5%* B. theta
			936 concordant pairs			
			106 discordant pairs			

Annotations: * value estimated as average of sensitivity and specificity, ¶ value based on leave-one-out analysis as reported by authors

Abbreviations: Transcriptional Unit (TU), Base Pair (bp), Specificity (Spe), Sensitivity (Sen).

A similar pattern of intergenic distances also emerged in *B. subtilis *(Table 3, Figure 7). We noted that many FP linkages had an intergenic distance <25 (46%) bases. The expansion of validated operon annotation in *E. coli *suggests these linkages for *B. subtilis *might actually be true positives which are currently annotated incorrectly.

Previous studies showed operon prediction improved when considering intergenic distance and whether proteins appeared in the same pathway [7,32,47]. We noted that 64% of predicted TP linkages with known COG classification for both proteins involved proteins in the same functional category; however only 22% of FP linkages were between proteins in the same functional category. While the combination of intergenic region and functional categories will improve specificity of operon prediction, the 36% of true positive linkages involving proteins not in the same functional category would be missed by a strategy requiring shared functional category.

## Discussion

The field of predicting protein-protein linkages has been active for more than two decades, but the application of computational methods for predicting protein linkages has only become popular in the past several years. Predicting which proteins interact in the cell is important in deciphering the function of proteins.

Computational approaches provide us with more information than the traditional homology approach, in which protein function is predicted based upon similarity to other proteins with known function. Even though the strict homology-based methods are effective for predicting protein functions of evolving close homologs, the methods perform poorly on distantly related proteins. Even a sophisticated homology-based method fails to successfully assign functions to all proteins of a particular organism [48]. Alternative approaches offer the benefit of being able to extract, combine, or compare information from a number of different sources, including information such as close physical location for a linkage in a genome, evolutionary conservation among multiple species, and similar association in different types of genomic context. These computational methods utilize the contexts in which the protein exists and thus can be useful in determining the role of many unclassified proteins.

We have looked at four genomic context computational methods for predicting protein functional linkages, comparing their performance on several prediction benchmarks. No one method dominated all benchmarks, in fact each method proved most effective for a distinct task. Gene neighborhood worked best for EcoCyc pathway reconstruction, Rosetta Stone for KEGG functional categories, and Phylogenetic profiles for COG functional categories.

The fourth method, Gene cluster (GC), is a conceptually simple method to identify potential operons. There are several other studies using computational methods to recognize regulatory elements in bacteria DNA (Table 4). Not all methods use the same definition of what constitutes a true positive so the quoted values are not strictly comparable and should be taken as indications only. Despite the particular differences, however, all methods tend to agree that intergenic distance alone allows approximately 82% recall. In addition to intergenic distance, these methods use a variety of other information, including codon usage statistics, gene expression data and regulatory features. The emphasis in these studies has been operon prediction in *E. coli *K12 and the transfer of methodology to other organisms has been met with mixed results. One study [14] found that an operon predictor based on intergenic distance in *E. coli *K12 worked equally well when applied to known operons of *B. subtilis*. Another previous study [7] reported 91% prediction accuracy when trained on *E. coli *K12 but accuracy reduced to 64% when tested on *B. subtilis*. One potential cause for the drop in performance lies in the incompleteness of the gold standard annotation source, as suggested by our results comparing different database versions. Figure 6, 7, together with Table 3, provide an immediate visual appreciation of the effect of database changes on reported results.

Our results showed GC was able to identify approximately 78% of the known operon pairs in *E. coli *K12 with 82% precision. We improved specificity of operon prediction by combining predicted linkages found by both GC and GN methods but to the detriment of sensitivity (Figure 6d). We also showed how the combination of intergenic and function information improved the accuracy of operon prediction. With these results on known operons, we also report a high percentage of unclassified proteins among linkages predicted by the GC method.

Another of the approaches, Gene neighborhood (GN), assumes that proteins located in a close neighborhood on the genome may have some functional commonalities. Such neighborhood relations occasionally are effective at predicting biological processes [16,18,20]. We reconstructed 48 known *E. coli *pathways using the predicted GN linkages. Our result is consistent with previous findings that GC or GN method had the highest AC (accuracy+coverage) using EcoCyc annotation than the other genomic context [38] (Figure 2d).

Rosetta Stone (RS) screens genomes for sequences that appear as two different proteins in one genome but are fused to create a single protein-chain in another genome. Fusion can facilitate kinetic coupling of consecutive enzymes in pathways or other forms of functional linkage between proteins. Our results showed that 30% of known membrane transporter proteins in KEGG (145 out of 489 proteins) are fused and 19% of the proteins in linkages found by RS method were membrane transporters (145 out of 776). Previous studies have shown that fusion events are common in Environmental information processing proteins [22]. The high coverage of the RS in Energy production and conversion (C), Amino acid transport and metabolism (E) and Signal transduction mechanism (T) proteins using COG annotation source is consistent with the Yanai *et al*. report [49] (Figure 2a). Previous findings confirm our result that RS method has the highest AC (accuracy+coverage) using the KEGG annotation source than the other genomic context [38] (Figure 2b). Previous studies have noted a high coverage of the RS method in metabolism proteins using KEGG annotation, and we also noted that the nucleotide metabolism proteins constitute one of the functional categories for which the RS method had a high coverage [38,50] (Figure 2b).

Our assessment showed a large percentage of linkages predicted by Phylogenetic profiles (PP) were assigned the same COG functional category. The stronger performance of PP over the other methods we suspect can be traced to the method of assigning COG function uniformly to all members of a given COG cluster and the strong presence of paralogs linked to PP. Nearly 28% of the linkages offered by PP were among proteins assigned to the same COG cluster identifier, which then received the same COG functional assignments, yet only 47% of these were assigned a common KEGG function. The ability to detect paralogs therefore may be the cause for the advantage of PP over the other methods in predicting COG function. Jothi *et al*., reported a high specificity in membrane transporter proteins when the reference set was composed of genomes from all three super-kingdoms (similar to our reference set) and this is consistent with our findings [27] (Figure 2b).

Using a network based approach for predicting protein function from neighbors in the network, we again found that RS was most successful at predicting KEGG functions while PP was most successful for COG function. Our results showed function predictions made using a weighted integration of linkages from methods greatly improved coverage while maintaining high sensitivity. The low overlap of linkages predicted by more than one method (<18%) did not allow strong discrimination among different weighting strategies though assignment of a single reliability value to all linkages from a method was slightly preferred over reliability assessments per linkage (Figure 4).

The methods of GC, GN, RS and PP all produced a large number of false positives. The main caveat of these methods is not always abundantly clear as to which linkages are real and which ones are false positives. Our results show these methods may not be an absolutely reliable predictor of a functional relation since there exists a large number of putative false positives which show linkage to functionally unrelated proteins. Nor can these methods differentiate among interacting and non-interacting homologs.

For any given method, without a filtering strategy, the high number of apparent false positives and false negatives is unavoidable However, as more detailed protein linkage databases become available, the total number of correctly identified linkage will rise while error rates will decrease. At this stage it is difficult to accurately assess specificity and sensitivity of computational methods because curated databases of genes and proteins are not available. As we showed, the precision of operon predictions increased from 47% to 82% by side-by-side comparison of two different releases of the RegulonDB database.

## Conclusion

Computational methods for protein-protein linkage have changed our perspective toward understanding proteins and their biological roles. They have provided an understanding of protein function intercellular sub-networks. Each of the most common methods, Gene cluster, Gene neighbor, Phylogenetic profiles, and Rosetta Stone have certain levels of effectiveness but they also have limitations that affect the reliability of functional interpretations of the linkages they predict. We used the latest available annotations and our results represent an update on previous findings and offer a number of new observations. Using several benchmarks we provided guidelines as to which method was most appropriate for a given prediction task. We demonstrated how changing the benchmark causes the dramatic difference on reported results.

## Methods

### Protein-protein linkages

Currently, several databases that compile functional linkages from genomic context predictions are available, including Prolinks [22], STRING [39], PLEX [23], Predictome [51]and more recently InPrePPI [38]. We used the Prolinks v2.0 database as a source of predictions because it implements all of the four functional prediction methods and is publicly available. Prolinks has better coverage than Predictome and unlike Predictome or PLEX, Prolinks uses a probabilistic scoring scheme to assign a confidence level to each functional linkage. The STRING and InPrePPI databases also provide probabilistic scores but STRING does not provide Gene cluster linkages and the Gene cluster definition in InPrePPI requires operon conservation across species which will miss operons which are genome specific [38].

We mapped accession IDs from Prolinks v2.0 predicted linkages to NCBI [1] and then to EcoCyc [30] for *E. coli *K12. An *E*-value of less than 10^-10 ^was used as the threshold for BLASTP in Prolinks to define a homolog of a query protein to be present in a secondary genome. In total, Prolinks lists 72,202 (27,339 unique) protein linkages for *E. coli *K12 using either the Gene cluster (GC), Gene neighbor (GN), Rosetta Stone (RS) or Phylogenetic profiles (PP) method (Table 1). Over one third of the linkages (9,623 out of 27,339 predicted linkages), were predicted with a confidence level of at least 0.6 using the Prolinks scoring scheme [22]. There were no predictions for GC above 0.7 or below 0.5 (Table 1). All reported results used the set of linkages found using the > 0.6 confidence level threshold.

### Generating the protein-protein linkage network

Networks were created and presented as graphs in which each protein is represented as a node and a linkage between proteins is represented by an edge. An edge existed between two nodes if the corresponding Prolinks linkage confidence score exceeded the threshold of 0.6 (Figure 1). For separation of connected components of the network and building the clusters of proteins, breadth-first search (BFS) graph algorithms were used. Network graphs were visualized using Cytoscape [52] an open-source, platform-independent environment for visualizing biological networks.

### Analyzing the topology of the network

The degree of a node in a graph is the number of edges connected to that node and nodes that are joined by an edge are said to be adjacent. A neighbor of a given node *i *is a node adjacent to *i*. The clustering coefficient *C *indicates the degree to which *k *neighbors of a particular node are connected to each other. Let *k*_*i *_be the number of neighbors of node *i *and *n*_*i *_be the number of edges in the network that exist among the neighbors of *i*. The clustering coefficient of node *i *was calculated as [40]

*C*_*i *_= 2*n*_*i*_/*k*_i_* (*k*_*i*_-1).

The average clustering coefficient was calculated by averaging *C *over all nodes *i*.

### Benchmark Annotation Sources

The accession IDs from predicted linkages were mapped to NCBI, and then to EcoCyc and KEGG. There were some disagreements about the number of proteins in *E. coli *K12: EcoCyc had entries for 4,449 proteins while the NCBI genome entry had 4,245 proteins, and 3,924 of these had a predicted linkage in Prolinks. While some proteins may have no predicted linkages by any method (reducing the number of proteins found in Prolinks), the discrepancy between EcoCyc and NCBI is unexplained. Pathways and operons defined in EcoCyc were sometimes supplemented with literature searches and with the NCBI, COG and KEGG databases, described below.

The NCBI Cluster of Orthologous Group (COG) [53] database classifies *E. coli *K12 proteins into the following 19 functional categories: Not classified (-); Energy production and conversion (C); Cell division and chromosome partitioning (D); Amino acid transport and metabolism (E); Nucleotide transport and metabolism (F); Carbohydrate transport and metabolism (G); Lipid metabolism (H); Coenzyme metabolism (I); Translation, ribosomal structure and biogenesis (J); Transcription (K); DNA replication, recombination and repair(L); Cell motility and secretion (N); Cell envelope biogenesis, outer membrane (M); Posttranslational modification, protein turnover, chaperones (O); Inorganic ion transport and metabolism (P); Secondary metabolites biosynthesis, transport and catabolism (Q); Signal transduction mechanism (T); Intracellular trafficking, secretion, and vesicular transport (U); and Defense mechanisms (V).

The Kyoto Encyclopedia of Genes and Genomes (KEGG) [41]database classifies proteins into four functional categories (19 sub-functional categories): **Cellular Processes **(Cell Motility (1); Cell Growth and Death (2)), **Environmental Information Processing **(Membrane Transport (3); Signal Transduction; (4)), **Genetic Information Processing **(Transcription (5); Translation (6); Folding, Sorting and Degradation (7); Replication and Repair (8)), and **Metabolism **(Carbohydrate Metabolism (9); Energy Metabolism (10); Lipid Metabolism (11); Nucleotide Metabolism (12); Amino Acid Metabolism (13); Metabolism of Other Amino Acids (14); Glycan Biosynthesis and Metabolism (15); Biosynthesis of Polypeptides and Non ribosomal Peptide (16); Metabolism of Cofactors and Vitamins (17); Biosynthesis of Secondary Metabolites (18); Xenobiotics Biodegradation and Metabolism (19)).

### Function Benchmark and Prediction Cross Validation

Two proteins were said to share the same function if they were assigned the same category by COG or KEGG. The percentage of linkages where the linked proteins shared the same function was calculated for each annotation source.

The set of classified proteins with at least one classified neighbor in each network (Phylogenetic profile, Rosetta Stone and Gene neighbor) was extracted to yield sets of size [946, 1,729 and 662] proteins by COG and [715, 1,213 and 459] proteins by KEGG for [PP, RS and GN] respectively (KEGG sub-category was equivalent to KEGG). The set of classified proteins was then divided uniformly at random into training (90%) and testing (10%) sets. The functions of the proteins in the training set were hidden and predicted from methods applied to the training set, and the percentage of correctly predicted function assignments to proteins was calculated over 100 cross validation splits. Predictions were made either sampling uniformly at random (UNIF) from the set of categories (COG 19 categories, KEGG 4 categories and KEGG 19 subcategories), or taking the function assigned to the majority of immediate neighbors (MAJOR) in the network (ties were broken randomly).

Comparisons between two methods were made by comparing the distribution of percent correct predictions from the 100 random runs of one method to another. Significance of the difference in means of these two distributions was measured using the two-sample t-test on the two sets of 100 values.

### Combinations of methods

We combined different sets of linkages predicted by GC, GN, RS or PP at Prolinks confidence 0.6. The overlap among all methods (AND) contained 20 linkages over 39 proteins and the set from at least one method (OR) contained 9,632 linkages over 3,323 proteins. The combined set of linkages predicted by GN, RS and PP at confidence level 0.6 (8,829 linkages among 2,574 proteins) was used in a cross validation setting for function prediction using a 90/10 split for training/testing over 100 random splits as before. Using this subset of linkages to define a network, we again compared the prediction method of assigning function uniformly at random (UNIF) against the majority assignment (MAJOR) prediction method. However, in this setting for MAJOR, we compared using the unweighted majority vote (OR) versus using a weighted vote where weights were assigned using the Noisy-Or model [39,44]with two schemes for assigning reliabilities. The Noisy-OR [39,44] method calculates a probability for each linkage (edge) *e *as:

$$pr\left(e\right)=1-\prod_{method}\left(1-r_{method}\right)$$

where the product ranges over all methods which predict the linkage. The reliability of a method *r*_*method *_was assigned by either of two strategies: Noisy-OR_conf_, where *r*_*method *_was the linkage confidence originally assigned by Prolink (ranging from 0.6 to 1.0 in this setting), and Noisy-OR_meth_, where *r*_*method *_was estimated as the average percent correct predictions from the cross-validation study using each method individually (Figure 3). For example, *r*_*RS *_= 75.28 for the KEGG subcategory gold standard, as given in Figure 3. The Noisy-OR_conf _strategy leveraged the confidence assessments of each linkage separately while the Noisy-OR_meth _strategy of assigning all linkages from a given method a single value was similar to that used in the STRING database [39]. The probability of the linkage was then used in calculating the weighted majority vote functional assignment (MAJOR) when considering the combination of linkages from the genomic context methods.

### Pathway Reconstruction

Each method was tested for its ability to reconstruct known *E. coli *pathways, attempting full coverage, by providing linkages connecting known members of the pathway. The pathway information was downloaded from EcoCyc[30]. There were 239 known pathways documented in EcoCyc, comprised of 2,029 proteins. Each pathway was given as a list of proteins annotated to the pathway. The 30 pathways that trivially contained only one protein were removed from further processing. The percentage of a pathway reconstructed under each method was calculated as the percentage of proteins annotated to the pathway that could be connected to at least one other member of the pathway through linkages predicted by the method.

### Operon Prediction Benchmark

To evaluate the correctness of protein linkages predicted by Gene cluster (GC), we obtained the set of experimentally verified *E. coli *K12 operons from RegulonDB [45], a database of transcriptional regulation and organization for *E. coli *K12. Experimental operons for *B. subtilis *were obtained from DBTBS [46]. The latest version of RegulonDB (June 2007) listed 2,684 transcription units (operons) in *E. coli *K12, but 1,853 of these were deemed trivial in that they contained only a single protein. The 831 multiprotein operons contained 2,654 proteins. The earlier version of RegulonDB (January 2006) listed 770 transcription units (operons) in *E. coli *K12, but 414 of these were deemed trivial in that they contained only a single protein. The 356 multiprotein operons contained 1,261 proteins. DBTBS (September 2007) listed 1,115 transcription units (operons), but 696 of these were deemed trivial in that they contained only a single protein. The 419 multiprotein operons contained 1,319 proteins. The intergenic distances between predicted protein linkage pairs by the Gene cluster method for *E. coli *K12 and *B. subtilis *were calculated using the NCBI annotations [1].

We evaluated the GC predicted linkages using Precision and Recall measures. Precision (also known as Positive Predictive Value PPV) was calculated as

Precision = TP/(TP+FP)

where we considered a true positive (TP) when both proteins involved in a linkage had a functional annotation in EcoCyc (DBTBS for *B. subtilis*) and were known to reside in the same operon by RegulonDB (DBTBS for *B. subtilis*) and a false positive (FP) when at least one of the classified proteins was not listed by RegulonDB (DBTBS) to be in the same operon. Recall (also known as Sensitivity) was calculated as:

Recall = TP/(TP +FN)

where the number of false negatives (FN) was the number of linkages missed among those existing in our gold standard (RegulonDB or DBTBS). The F-measure was then calculated as

F-measure = (2* Recall*Precision)/(Recall + Precision)

## Authors' contributions

AKF conceived of the project and implemented the methods. SML and AKF analyzed and interpreted the results. The manuscript was written by AKF and SML and edited by RTG and LH. RTG oversaw all biological aspects of the work and LH supervised the computational aspects.
